# Identification of Multiple Cracks in Composite Laminated Beams Using Perturbation to Dynamic Equilibrium

**DOI:** 10.3390/s21186171

**Published:** 2021-09-15

**Authors:** Aimin Deng, Maosen Cao, Qitian Lu, Wei Xu

**Affiliations:** 1 Department of Engineering Mechanics, Hohai University, Nanjing 210098, China; dengaimin@hhu.edu.cn (A.D.); cmszhy@hhu.edu.cn (M.C.); qtlu@hhu.edu.cn (Q.L.); 2 Jiangsu Province Wind Power Structural Engineering Research Center, Hohai University, Nanjing 210098, China

**Keywords:** multiple crack identification, composite laminated beam, perturbation to dynamic equilibrium, non-contact vibration measurement, laser scanning

## Abstract

Identification of cracks in beam-type components is significant to ensure the safety of structures. Among the approaches relying on mode shapes, the concept of transverse pseudo-force (TPF) has been well proved for single and multiple crack identification in beams made of isotropic materials; however, there is a noticeable gap between the concept of TPF and its applications in composite laminated beams. To fill this gap, an enhanced TPF approach that relies on perturbation to dynamic equilibrium is proposed for the identification of multiple cracks in composite laminated beams. Starting from the transverse equation of motion, this study formulates the TPF in a composite laminated beam for the identification of multiple cracks. The capability of the approach is numerically verified using the FE method. The applicability of the approach is experimentally validated on a carbon fiber-reinforced polymer laminated beam with three cracks, the mode shapes of which are acquired through non-contact vibration measurement using a scanning laser vibrometer. In particular, a statistic manner is utilized to enable the approach to be feasible to real scenarios in the absence of material and structural information; besides, an integrating scheme is utilized to enable the approach to be capable of identifying cracks even in the vicinity of nodes of mode shapes.

## 1. Introduction

Initial cracks in beam-type structural components can develop to significant extents subject to long-term transverse loads, jeopardizing local stiffness against bending moments, by which structural failures can be induced [1]. Thereby, approaches for identifying cracks in beam-type structural components are worth investigating, especially multiple crack identification, which is more challengeable compared with single crack identification [2]. Compared with approaches relying on natural frequencies [3,4,5,6,7,8,9], approaches relying on mode shapes are naturally capable of locating cracks because they contain spatial locations of structural elements.

In the recent two decades, approaches using curvature mode shapes (CMSs) have been widely developed for the identification of multiple damage in beams. Lin and Cheng [10] utilized CMSs for the identification of double cracks in beams. Dawari and Vesmawala [11] identified double cracks in beams by the difference between a pair of CMSs under damaged and intact statuses. Rising from pairs of damaged and intact mode shapes, Abdel Wahab and De Roeck [12] identified multiple damage in a concrete bridge by averaged difference of CMSs. Sung et al. [13] used the normalized curvature of a uniform load surface for the identification of multiple damage in beams. Cao et al. [14] proposed the complex-wavelet CMS for the identification of multiple cracks in composite laminated beams. More recently, the Teager–Kaiser energy (TKE) method has been proposed for the identification of multiple cracks in beams [15]. The proposed TKE method was further applied to composite laminated beams [16]. A novel concept of modal Teager–Kaiser energy was proposed for the identification of multiple cracks using high-order mode shapes of Timoshenko beams [17]. The experimental results suggest that the TKE method is much more sensitive to cracks than the CMS method. The pseudo-force (PF) approach is another approach for structural damage identification relying on mode shapes, which has attracted increasing attention in the recent decade. Superior to other approaches relying on mode shapes, the PF appears at damaged locations only and vanishes at intact locations, by which the occurrence and locations of multiple cracks in a beam can be characterized by the peaks of the PF. Inspired by the idea of reconstructing the external force applied on a beam component [18], the concept of transverse PF (TPF) was proposed by canvassing the local perturbation to transverse dynamic equilibrium of beam components [19]. To enhance the noise robustness of the PF approach, Cao et al. [20,21] proposed the concept of multi-scale TPF by integrating multi-scale analysis into the TPF. For the same purpose, the “weak formulation” of the TPF was developed by introducing weighted integration to form the “region-by-region” inspection manner [22,23]. For the identification of multiple damage, integrating schemes were developed by fusing TPFs that arise from multiple flexural mode shapes [24,25].

Although the concept of TPF has been widely investigated for single and multiple crack identification in beams made of isotropic materials, its applications in composite laminated beams are hindered owing to the absence of prior knowledge of complex structural and material parameters. To fill this noticeable gap between the concept of TPF and identification of cracks in composite laminated beams, an enhanced approach is proposed in this study, which relies on perturbation to the dynamic equilibrium of a composite laminated beam. In particular, the approach is baseline-free, which is independent of structural and material parameters of composite laminated beams.

The rest of the paper is organized as follows. Starting from the transverse equation of motion, Section 2 formulates the TPF in a composite laminated beam for the identification of multiple cracks. Section 3 numerically verifies the capability of the approach by the finite element (FE) method. Section 4 experimentally validates the applicability of the approach on a laminated carbon fiber-reinforced polymer (CFRP) beam with three cracks. In particular, the mode shapes of the beam are acquired through non-contact vibration measurement using a scanning laser vibrometer (SLV). Section 5 presents concluding remarks.

## 2. Identification of Multiple Cracks Using Perturbation to Dynamic Equilibrium

Consider a symmetric fiber-reinforced laminated beam, which consists of M thin plies that can be regarded as homogeneous and orthotropic. The transverse equation of motion of the laminated beam subject to a transverse excitation f(x,t) is written as [26]

(1) $$D_{11}\frac{\partial^{4}w(x,t)}{\partial x^{4}}+\rho \frac{\partial^{2}w(x,t)}{\partial t^{2}}=f(x,t),$$

where w(x,t) is the transverse displacement, $D_{11}=\frac{1}{3}\sum_{d=1}^{M}b{\bar{Q}}_{11}^{\left(d\right)}(z_{d}^{3}-z_{d-1}^{3})$ is the stiffness coefficient with b being the width of the beam, ${\bar{Q}}_{11}^{\left(d\right)}$ is the material coefficient of the *d*th ply, $z_{d}$ is the distance from the middle surface to the surface of the *d*th ply having the furthest *z*-coordinate in the thickness direction, and $\rho =\sum_{d=1}^{M}b\rho^{\left(d\right)}(z_{d}-z_{d-1})$ is the average mass density of the laminated beam per unit length and $\rho^{\left(d\right)}$ being the density of the *d*th lamina per unit volume.

Considering a composite laminated beam that bears multiple cracks, one can assume the stiffness coefficients and average mass density at intact locations as $D_{11}^{I}$ and $\rho^{I}$, respectively, and $D_{11,i}^{C}$ and $\rho_{i}^{C}$ at the location of the *i*th crack. Superscripts I and C indicate intact and cracked statuses, respectively.

(2) $$D_{11}(x)=\{\begin{array}{ll} D_{11}^{I} & x\neq x_{i}\\ D_{11,i}^{C}(x) & x=x_{i} \end{array},$$

(3) $$\rho (x)=\{\begin{array}{ll} \rho^{I} & x\neq x_{i}\\ \rho_{i}^{C}(x) & x=x_{i} \end{array}.$$

**Figure 1:**
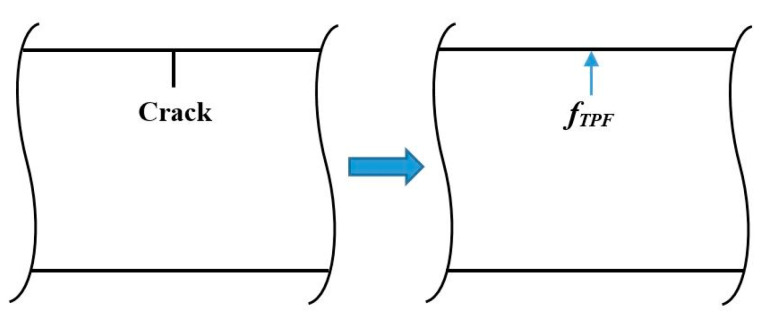
Sketch of crack-induced TPF.

Thereby, Equation (1) can be rearranged as

(4) $$D_{11}^{I}\frac{\partial^{4}w(x,t)}{\partial x^{4}}+\rho^{I}\frac{\partial^{2}w(x,t)}{\partial t^{2}}=f(x,t)+f_{TPF}(x,t),$$

where $f_{TPF}(x,t)$ denotes the crack-induced equivalent transverse force, defined as the TPF (illustrated in Figure 1), which exists at the crack locations only and vanishes at intact locations:

(5) $$f_{TPF}(x,t)=\{\begin{array}{ll} 0 & x\neq x_{i}\\ {}[D_{11}^{I}-D_{11,i}^{C}(x)]\frac{\partial^{4}w(x,t)}{\partial x^{4}}+[\rho^{I}-\rho_{i}^{C}(x)]\frac{\partial^{2}w(x,t)}{\partial t^{2}} & x=x_{i} \end{array}.$$

Regarding the elements of the composite laminated beam that bears no transverse excitation, i.e., f(x,t)=0, Equation (4) becomes

(6) $$D_{11}^{I}\frac{\partial^{4}w(x,t)}{\partial x^{4}}+\rho^{I}\frac{\partial^{2}w(x,t)}{\partial t^{2}}=f_{TPF}(x,t).$$

One can obtain the *n*th transverse mode shape $W_{n}(x)$ by assuming $w(x,t)=W_{n}(x)\sin\omega_{n}t$, where $\omega_{n}$ denotes an undamped natural frequency of the beam associated with the *n*th transverse mode. Substituting $w(x,t)=W_{n}(x)\sin\omega_{n}t$ into Equation (6) yields

(7) $$D_{11}^{I}\frac{d^{4}W_{n}(x)}{dx^{4}}-\omega_{n}^{2}\rho^{I}W_{n}(x)=F_{TPF}(x),$$

where $F_{TPF}(x)$ denotes the amplitude of $f_{TPF}(x,t)$:

(8) $$F_{TPF}(x)=\{\begin{array}{ll} 0 & x\neq x_{i}\\ \left[D_{11}^{I}-D_{11,i}^{C}(x)]\frac{d^{4}W_{n}(x)}{dx^{4}}-\omega_{n}^{2}[\rho^{I}-\rho_{i}^{C}(x)]W_{n}(x\right) & x=x_{i} \end{array}.$$

Equation (7) indicates that the amplitude of the TPF is associated with reductions of the stiffness and mass of the beam elements that bear cracks. Thereby, in this study, the absolute value of $F_{TPF}(x)$ is used to establish a damage index (DI), denoted as $DI_{n}(x)$, by which the occurrence and location of the cracks can be characterized:

(9) $$DI_{n}(x)=\left|D_{11}^{I}\frac{d^{4}W_{n}(x)}{dx^{4}}-\omega_{n}^{2}\rho^{I}W_{n}(x)\right|.$$

To eliminate noise interference caused by noise components involved in measured mode shapes, multiscale analysis [27] is utilized to enhance the robustness of the DI against environmental noise interference. Instead of the conventional “point-by-point” manner for inspecting measured mode shapes, the “region-by-region” manner is employed in this study. In particular, a scaled Gaussian windowing function ${\bar{g}}_{s}(x)$ slides along a mode shape to produce multi-scale mode shapes, denoted as $W_{n,s}(x)$ [28]:

(10) $$\bar{W_{n}}(v,s)=\frac{1}{\sqrt{s}}W_{n}⊗{\bar{g}}_{s}(v),$$

(11) $$W_{n,s}(x)={\bar{W_{n}}(v,s)|}_{v=x},$$

where ${\bar{g}}_{s}(x)=\frac{1}{\sqrt{s}}g(\frac{-x}{s})$ with $g(x)={\left(2/\pi \right)}^{1/4}e^{-x^{2}}$ being the Gaussian function; s is the scale parameter, which is adjustable; and ⊗ denotes convolution.

A multi-scale DI (MDI), denoted as $DI_{n,s}(x)$, is established by replacing W(x) in Equation (9) with $W_{n,s}(x)$:

(12) $$DI_{n,s}(x)=\left|D_{11}^{I}\frac{d^{4}W_{n,s}(x)}{dx^{4}}-\omega_{n}^{2}\rho^{I}W_{n,s}(x)\right|.$$

As the adjustable scale parameter is integrated into the MDI, the MDI exhibits an intrinsic multi-scale property, which has two merits [28]. When the scale parameter gradually increases, the ever-wider Gaussian window can average random noise components, leading to noise-free mode shapes. On the other hand, crack-induced singularity peaks in the MDI are naturally retained with the increasing scale parameter, by which cracks can be indicated and located. Thereby, MDIs are suitable for identifying cracks in composite laminated beams in noisy conditions owing to the multi-scale property.

Although the strategy of crack identification using the TPF is simple and straightforward, material and structural parameters of the vibrating structures under inspection are required, which hinders its applications to in-service structures whose material and structural parameters are unavailable. Addressing this problem, Equation (12) is normalized by means of dividing Equation (12) by $D_{11}^{I}$, whereby a normalized MDI (NMDI) can be obtained:

(13) $$DI_{n,s}^{*}(x)=\left|\frac{d^{4}W_{n,s}(x)}{dx^{4}}-\lambda^{4}W_{n,s}(x)\right|,$$

where $\lambda^{4}=\omega_{n}^{2}\rho^{I}/D_{11}^{I}$ is a constant related to material and structural parameters of a composite laminated beam. As material and structural parameters are usually unknown, it is almost impossible to directly determine the constant $\lambda^{4}$. In this study, because $DI_{n,s}^{*}(x)$ in Equation (13) almost vanishes at intact locations of the beam, $\lambda^{4}[x]$ at intact locations can be calculated in a statistic manner [26]:

(14) $$\lambda^{4}[x]=\frac{d^{4}W_{n,s}}{dx^{4}}[x]/W_{s}[x].$$

The pointwise constant $\lambda^{4}[x]$ with the maximum probability can be approximately regarded as the constant $\lambda^{4}$. It is noteworthy that the amplitudes of the crack-induced singularity peaks in $DI_{n,s}^{*}(x)$ heavily depend on the crack locations. Some modes are sensitive to cracks, whereas some are insensitive. As it is well known that displacements at nodes always vanish, cracks in the vicinity of nodes of the mode shapes can hardly produce singularity peaks with noticeable amplitudes. With this concern, this study utilizes an integrating scheme for multiple crack identification of composite laminated beams by fusing multiple modes [22]:

(15) $$DI_{s}^{*}(x)=\sum_{n}DI_{n,s}^{*}(x).$$

## 3. Numerical Verification

### 3.1. Numerical Model

**Figure 2:**
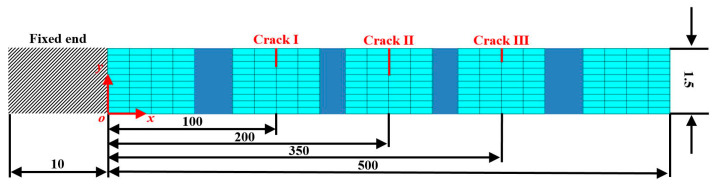
Numerical model of the CFRP laminated beam with three cracks, the dimensions of which are in millimeters (the blue sections are used to display the intact segments of the beam).

**Figure 3:**
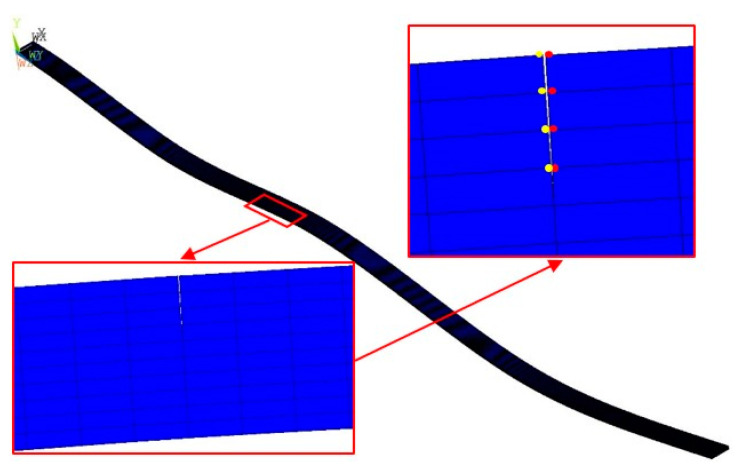
The fourth mode shape of the cracked beam with a zoomed-in view of Crack II (nodes on two crack interfaces are marked by red and yellow dots).

A five-ply CFRP cantilever beam is considered as a numerical specimen. The length, width, and thickness of the beam are 510 mm, 10 mm, and 1.5 mm, respectively. The thickness of each ply is 0.3 mm and orientations of the five plies are [0/45/90/−45/0°]. A numerical model of the beam is shown in Figure 2 with dimensions in millimeters. Note that the blue sections are used to display the intact segments of the beam as the length-to-thickness ratio of the beam is large. The fixed end of the cantilever beam spans 10 mm along the beam from the left edge of the beam. The first, second, and third cracks (denoted as Crack I, II, and III, respectively) are modeled at locations 110 mm, 210 mm, and 360 mm from the left edge, respectively. Crack I is 4.5 mm deep, which reaches the middle of the first and second plies; Crack II is 6 mm deep, which goes through the first two plies; and Crack III is 3 mm deep, which goes through the first ply. The CFRP laminated beam is modeled by the FE software ANSYS with eight-node hexahedral elements whose dimensions are 0.5 mm × 0.5 mm × 0.15 mm in the length, width, and thickness directions, respectively. The elastic moduli $E_{11}$ and $E_{22}$ in the length and width directions are 92 GPa and 8 GPa, respectively; in-plane shear modulus $G_{12}$, Poisson’s ratio $\nu_{12}$, and material density ρ are 2.9 GPa, 0.33, and 1400 Kgm^−3^, respectively. The cracks are modeled by inserting non-thickness interfaces between crack interfaces, on which the coincident nodes in adjacent but separated elements are distributed. Mode shapes can be calculated through the modal analysis module in the software and only flexural mode shapes are utilized in this study. Figure 3 shows the fourth mode shape of the CFRP laminated beam with a zoomed-in view of Crack II, on the two interfaces of which nodes are marked in different colors (yellow and red) for distinction. Without loss of generality, by introducing the dimensionless coordinates ζ=x/500, Crack I, II, and III are located at $\zeta_{1}=0.2$, $\zeta_{2}=0.4$, and $\zeta_{3}=0.7$, respectively.

### 3.2. Numerical Results

**Figure 4:**
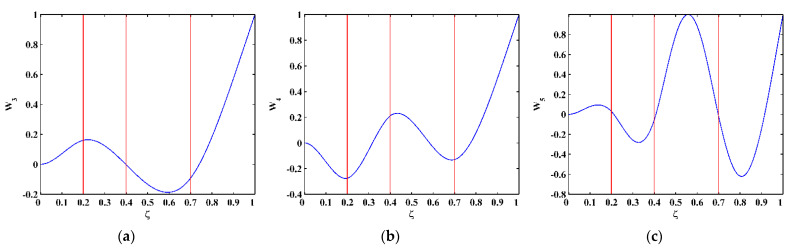
The (**a**) third, (**b**) fourth, and (**c**) fifth mode shapes (locations of cracks are indicated by red lines).

**Figure 5:**
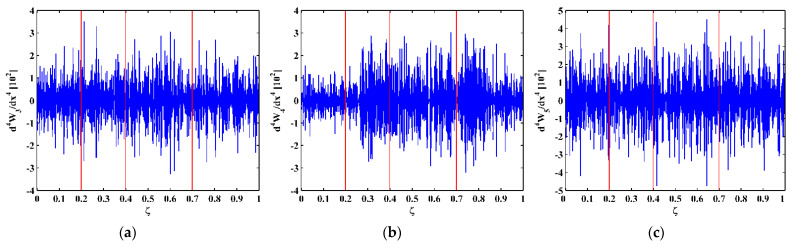
Derivatives of the (**a**) third, (**b**) fourth, and (**c**) fifth mode shapes (locations of cracks are indicated by red lines).

**Figure 6:**
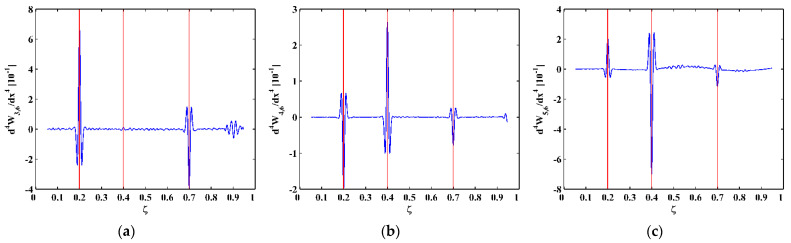
Derivatives of the (**a**) third, (**b**) fourth, and (**c**) fifth multi-scale mode shapes (locations of cracks are indicated by red lines).

As higher-order mode shapes are more sensitive to local damage [17], the third, fourth, and fifth mode shapes of the CFRP laminated beam are selected in this study for identification of multiple cracks. Displacement magnitudes of 1001 nodes along the line in the middle of the intact side (510 mm in length and 10 mm in width) of the beam are extracted to represent the third, fourth, and fifth mode shapes, as shown in Figure 4a–c, respectively. In Figure 4, three vertical red lines for each mode shape are plotted to mark locations of Crack I, II, and III. It is noteworthy that Crack II happens to lie in one node of the third mode shape (Figure 4a) and all the three cracks are very closed to the nodes of the fifth mode shape (Figure 4c). To simulate environmental noise contamination during the measurement, white Gaussian noise is added to produce mode shapes with the signal-to-noise ratio (SNR) of 60 dB. By the finite difference method, the fourth-order derivatives of mode shapes in Equation (9) are calculated and shown in Figure 5. Noise components are amplified in the derivatives owing to the differentiation and dominate the derivatives, masking any damage features. To deal with noise interference, the mode shapes are processed using multi-scale analysis as per Equations (10) and (11), from which multi-scale mode shapes are obtained. By gradually increasing the parameter scale to a satisficing level of six for the satisfying results with the minimum scale parameter, noise components in mode shapes can be basically eliminated, leading to the relatively smooth derivatives of the multi-scale mode shapes, as shown in Figure 6.

**Figure 7:**
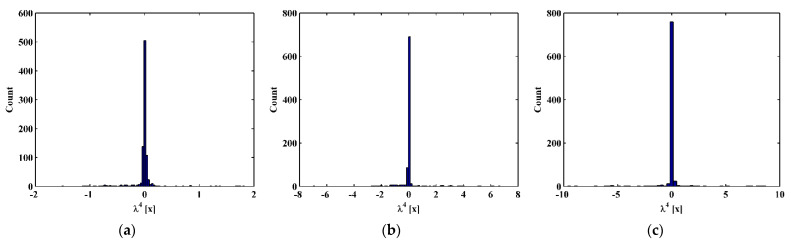
Distributions of constants $\lambda^{4}[x]$ for the (**a**) third, (**b**) fourth, and (**c**) fifth mode shapes.

**Figure 8:**
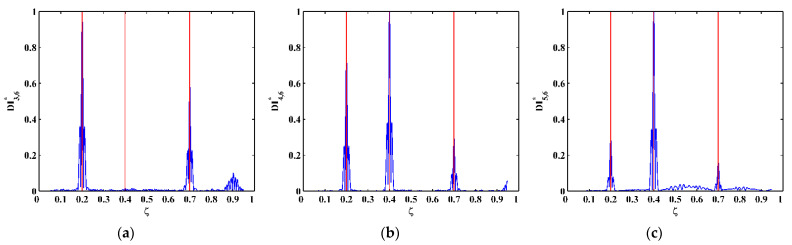
NMDIs for the (**a**) third, (**b**) fourth, and (**c**) fifth mode shapes (locations of cracks are indicated by red lines).

**Figure 9:**
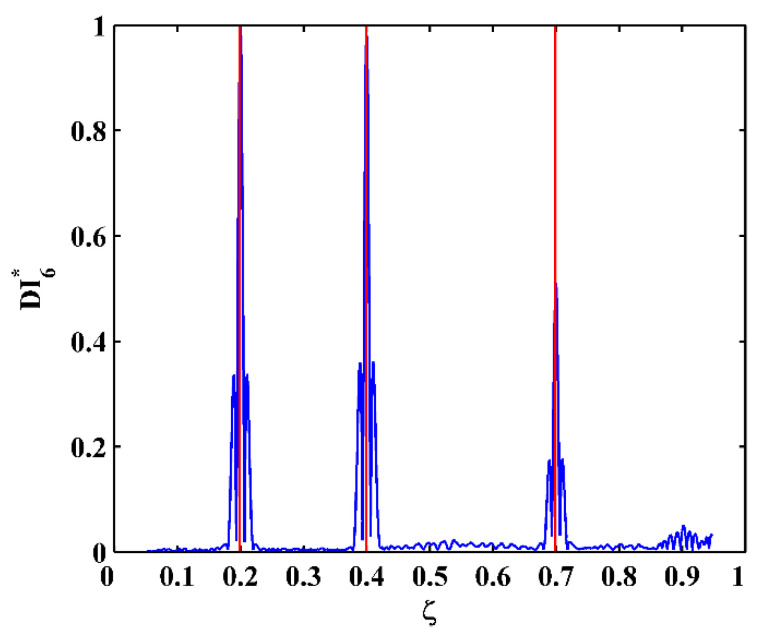
Fused NMDI for multiple crack identification (locations of cracks are indicated by red lines).

By substituting $d^{4}W_{n,s}/dx^{4}[x]$ and $W_{n,s}[x]$ for each measurement point to Equation (14), one can obtain pointwise constants $\lambda^{4}[x]$, whose distributions in 100 uniform sections are illustrated in Figure 7a–c for the third, fourth, and fifth mode shapes, respectively. In Figure 7, distributions of $\lambda^{4}[x]$ are concentrated around the sections with the maximum counts, the mean values of which can be approximately regarded as the constants $\lambda^{4}$ in a statistic perspective. Thereby, the constants $\lambda^{4}$ can be estimated in a statistic manner by finding the intervals of pointwise constants $\lambda^{4}[x]$ with the maximum counts. By substituting the estimated $\lambda^{4}$ to Equation (13), one can obtain the NMDI $DI_{n,s}^{*}$ for the three mode shapes, as shown in Figure 8. Without loss of generality, each $DI_{n,s}^{*}$ is normalized by making its maximum value being unit. It can be seen from Figure 8 that singularity peaks appear and sharply rise in each $DI_{n,s}^{*}$ to indicate the occurrence and locations of the cracks, because the TPFs exist at crack locations and vanish at intact locations of the beam. However, some peaks are noticeable, whereas others are less pronounced; in Figure 8a, no peak appears at the location of Crack II for the third mode shape owing to the node effect. To display all identified cracks, the integrating scheme is utilized as per Equation (15), by which $DI_{n,s}^{*}$ for the three mode shapes are averaged to produce the fused NMDI $DI_{s}^{*}$. As shown in Figure 9, three singularity peaks evidently indicate the occurrence of Crack I, II, and III; besides, it can be clearly seen that the identified cracks are located at $\zeta_{1}=0.2$, $\zeta_{2}=0.4$, and $\zeta_{3}=0.7$, respectively, which corresponds to the actual locations of the three cracks indicated by the three red lines.

**Figure 10:**
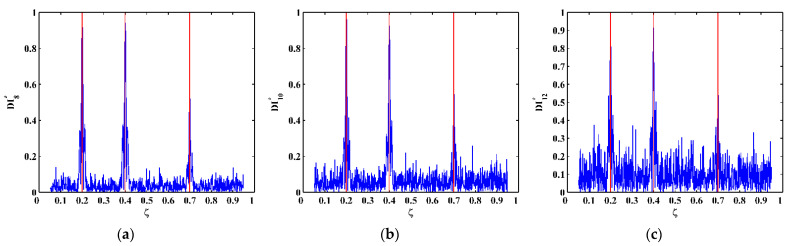
Fused NMDIs associated with SNRs of (**a**) 50, (**b**) 40, and (**c**) 30 dB (locations of cracks are indicated by red lines).

To explore the ultimate tolerance of the approach against noise interference, SNRs of the mode shapes gradually decrease to 50, 40, and 30 dB to simulate increasingly intensive noise levels. The corresponding fused NMDIs associated with scale parameters 8, 10, and 12 are shown in Figure 10a–c, respectively. It can be seen from Figure 10a,b that, although noise interference increases to cause burrs in the fused NMDIs, three singularity peaks in each fused NMDI can still evidently pinpoint the three cracks. Nevertheless, in Figure 10c, the singularity peak for the third crack is largely affected by the peaks caused by noise interference, becoming ambiguous for the identification of crack. Thereby, the noise level of SNR 30 dB can be regarded as the ultimate noise tolerance of the approach for the numerical scenarios in this study.

## 4. Experimental Validation

### 4.1. Experimental Specimen and Setup

**Figure 11:**
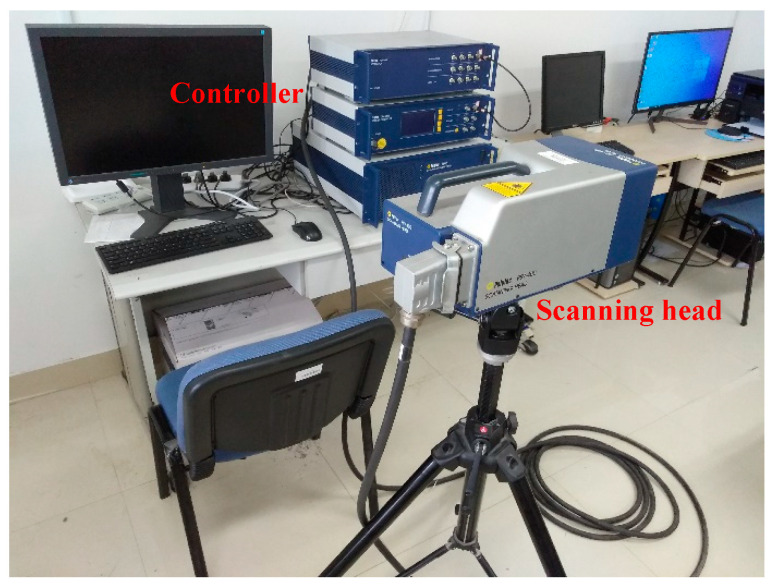
SLV system.

**Figure 12:**
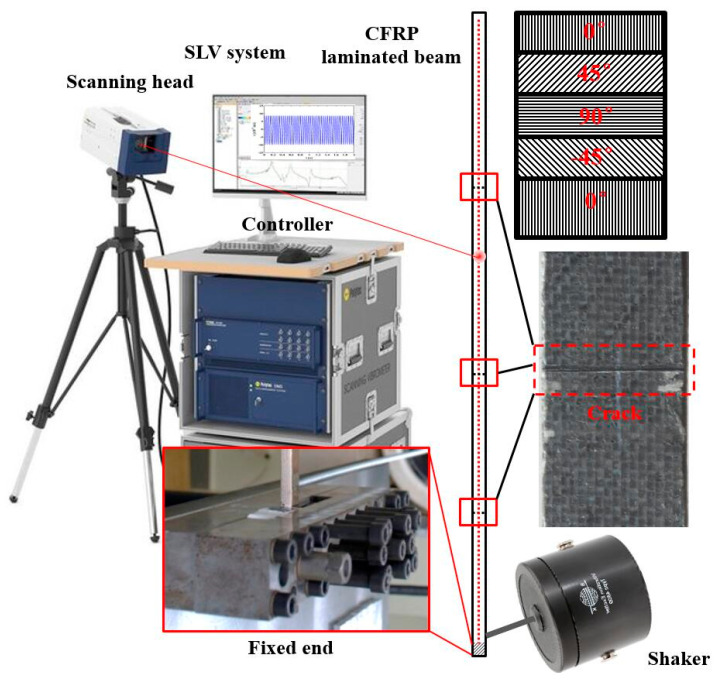
Schematic of experimental vibration measurement.

The experimental specimen is a CFRP cantilever beam that consists of five plies. The dimensions of the beam are 500 mm, 10 mm, and 1.5 mm in the length, width, and thickness directions, respectively. Each ply is 0.3 mm in thickness and the orientations of the five plies are [0/45/90/−45/0°]. Spanning 10 mm along the beam from the edge of the left end, the beam is fixed by a vice. The first, second, and third cracks (also denoted as Crack I, II, and III, respectively) were manufactured using a very thin knife at locations 113 mm, 221 mm, and 365 mm from the left edge, respectively. Both Crack I and Crack II are about 3–6 mm deep, which reaches between the first and second plies; Crack III is about 2–3 mm deep, which is in the first ply. On the cracked side of the beam, a vibration shaker (B&K 4809, B&K, Nærum, Denmark) is attached at 15 mm from its left edge as an actuator to generate single-tone harmonic excitations. When the beam vibrates under the harmonic excitations at the natural frequencies, an SLV (PSV-400, Polytec, Waldbronn, Germany) is used as a sensor to scan the intact side of the beam, whereby the steady-state responses can be acquired from every single measurement point. The SLV system is shown in Figure 11. The measurement line lies on the intact side (500 mm in length and 10 mm in width) of the beam, which spans 10 mm to 496 mm from the left edge; 499 measurement points are uniformly distributed along the measurement line. The dimensionless locations for the first, second, and third cracks at the measurement line are $\zeta_{1}=0.212$, $\zeta_{2}=0.434$, and $\zeta_{3}=0.730$, respectively. For clear illustration, schematic of the experimental vibration measurement is shown in Figure 12.

### 4.2. Experimental Results

**Figure 13:**
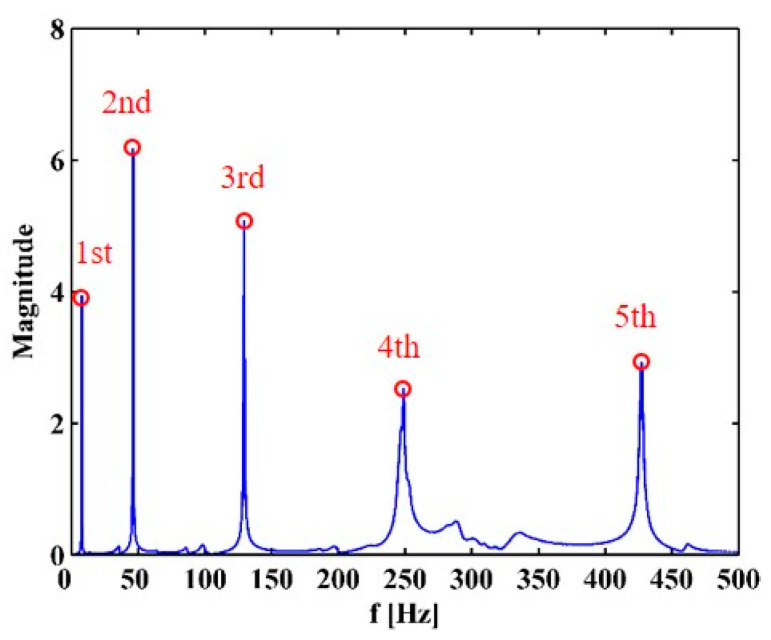
FRF of the CFRP laminated beam (the first five natural frequencies are marked by red dots).

**Figure 14:**
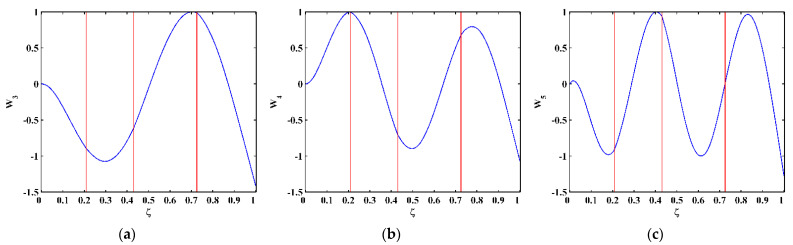
The (**a**) third, (**b**) fourth, and (**c**) fifth mode shapes (locations of cracks are indicated by red lines).

**Figure 15:**
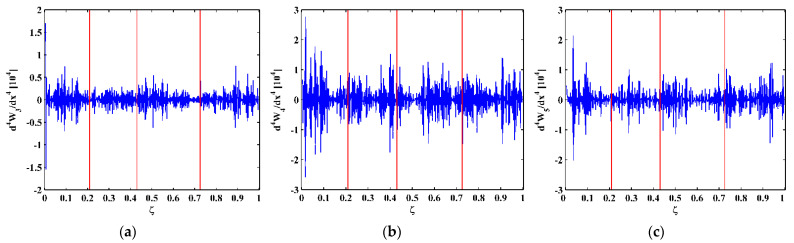
Derivatives of the (**a**) third, (**b**) fourth, and (**c**) fifth mode shapes (locations of cracks are indicated by red lines).

**Figure 16:**
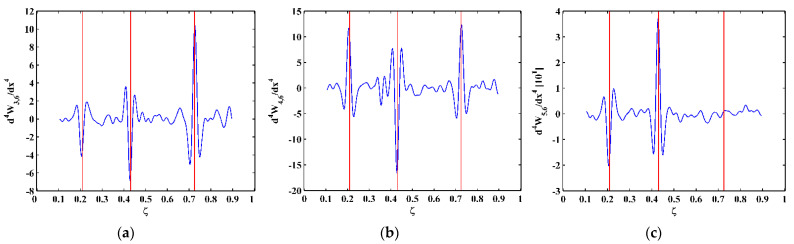
Derivatives of the (**a**) third, (**b**) fourth, and (**c**) fifth multi-scale mode shapes (locations of cracks are indicated by red lines).

Modal analysis is performed on the CFRP laminated beam over a wide range of frequencies [29], by which the first five flexural modes of the beam are found. The velocity response close to the free end of the beam is acquired by the SLV, from which the frequency response function (FRF) is obtained. The first five natural frequencies of the beam can be determined as 7.4, 45.9, 129.2, 248.8, and 427.2 Hz by the peaks in the magnitudes of the FRFs (marked by red dots), as shown in Figure 13. The operating deflection shapes (ODSs) of the beam at the third, fourth, and fifth natural frequencies are obtained by picking up values of real parts of ODSs at each measurement point. For this lightly-damped beam, the ODSs associated with the natural frequencies can be regarded as the corresponding mode shapes [30]. The third, fourth, and fifth mode shapes, as shown in Figure 14, are selected for the identification of multiple cracks in this study. Note that Crack III happens to lie in one node of the fifth mode shape. As expected, Figure 15 shows that noise interference masks damage features in the derivatives of the mode shapes. To deal with this problem, multi-scale analysis is performed on the mode shapes by Equations (10) and (11) for de-noising, by which the noise interference can be basically removed when the parameter scale increases to six. Hereby, the relatively smooth derivatives of the multi-scale mode shapes are obtained and shown in Figure 16.

**Figure 17:**
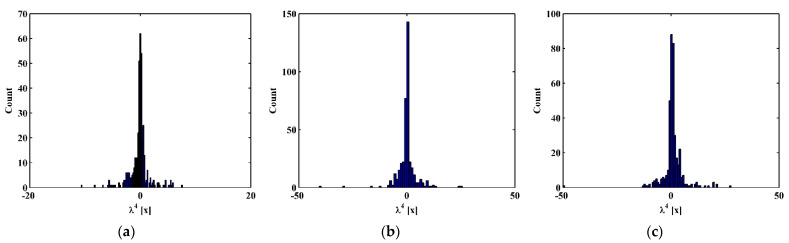
Distributions of constants $\lambda^{4}[x]$ for the (**a**) third, (**b**) fourth, and (**c**) fifth mode shapes (locations of cracks are indicated by red lines).

**Figure 18:**
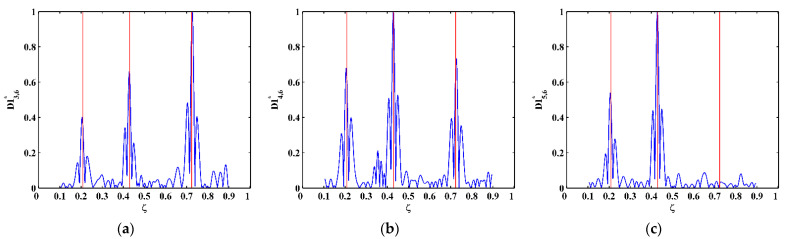
NMDIs for the (**a**) third, (**b**) fourth, and (**c**) fifth mode shapes (locations of cracks are indicated by red lines).

**Figure 19:**
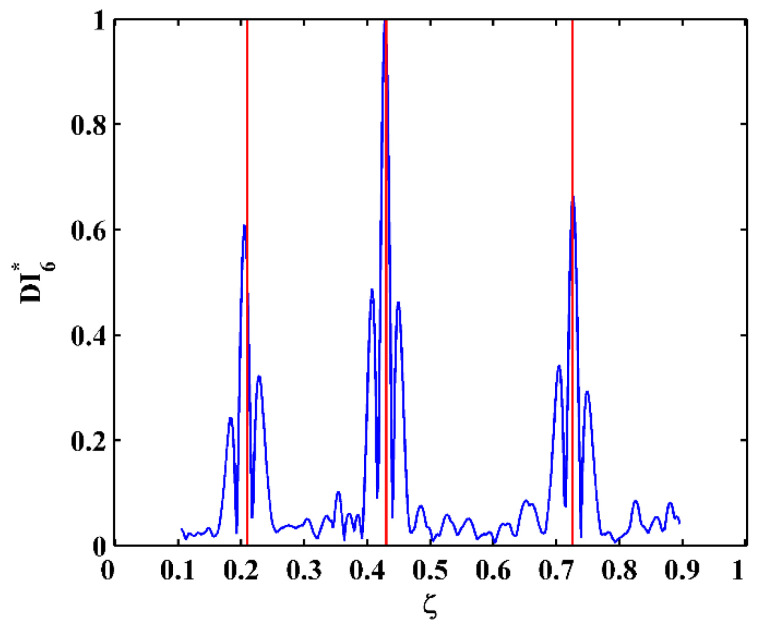
Fused NMDI for multiple crack identification (locations of cracks are indicated by red lines).

One can obtain pointwise constants $\lambda^{4}[x]$ for each measurement point by substituting $d^{4}W_{n,s}/dx^{4}[x]$ and $W_{n,s}[x]$ into Equation (14). Distributions of $\lambda^{4}[x]$ in 100 uniform sections are illustrated in Figure 17a–c for the third, fourth, and fifth mode shapes, respectively. The mean values of the intervals of the $\lambda^{4}[x]$ with the maximum counts can be regarded as $\lambda^{4}$ and substituted to Equation (13), by which the corresponding NMDI $DI_{n,s}^{*}$ can be obtained and shown in Figure 18. The same as those in the numerical simulation, each $DI_{n,s}^{*}$ is normalized with its maximum value being unit. It can be seen from Figure 18 that the occurrence of the cracks can be manifested by the singularity peaks; besides, the cracks can be pinpointed by the locations of the singularity peaks. However, as shown in Figure 18c, no peak appears at the location of Crack III because the crack happens to be located at one node of the fifth mode shape. By Equation (15), the integrating scheme is utilized to fuse $DI_{n,s}^{*}$ that are associated with the three mode shapes. The fused NMDI $DI_{s}^{*}$ is produced and shown in Figure 19, where all cracks are pinpointed at about $\zeta_{1}=0.21$, $\zeta_{2}=0.43$, and $\zeta_{3}=0.73$, in good agreement with the actual locations of Crack I, II, and III (indicated by the red lines), respectively.

## 5. Concluding Remarks

Single and multiple crack identification in beams has been well investigated using the TPF approach, nevertheless, the applicability of the TPF to composite laminated beams is hindered owing to the nature of the concept of TPF, which is formulated from the perturbation to dynamic equilibrium of elements in beams made of isotropic materials. To fill this gap, the TPF approach is enhanced in this study for the identification of multiple cracks in composite laminated beams. Starting from the transverse equation of motion of a composite laminated beam, its TPF is formulated for crack identification. The capability of the approach is numerically verified using the FE method and its applicability is experimentally validated through non-contact vibration measurement using an SLV. Some conclusions are as follows.

(1) The TPF exists at the crack locations only and vanishes at intact locations, based on which the DI is established using the absolute value of the amplitude of the TPF. The occurrence of multiple cracks can be manifested by the singularity peaks in the DI. Furthermore, cracks can be pinpointed by the locations of the peaks.
(2) To enhance the robustness of the DI against noise interference, multi-scale analysis is integrated into the DI. By sliding a scaled Gaussian window function along a mode shape signal, the “region-by-region” manner is utilized to average noise components in the noisy mode shape. On the other hand, crack-induced singularity peaks in the MDIs are naturally retained for the identification of cracks.
(3) To deal with unknown material and structural parameters that are required to formulate TPFs in composite laminated beams, a statistic manner is utilized to estimate the constants of the TPFs related to the material and structural parameters. In this regard, the proposed approach is a baseline-free approach, feasible to real scenarios in the absence of material and structural information.
(4) To remove the node effect that hinders the identification of multiple cracks in beams, an integrating scheme is utilized to fuse identification results associated with multiple mode shapes, whereby even cracks in the vicinity of nodes of mode shapes can be evidently identified.

## Data Availability

Not applicable.
